# Structural Variants Associated with Sporadic Alzheimer's Disease Pathologies and Segregates in Caribbean Hispanics and Non‐Hispanic White Families

**DOI:** 10.1002/alz70855_107342

**Published:** 2025-12-24

**Authors:** Tamil Iniyan Gunasekaran, Dolly Reyes‐Dumeyer, André Corvelo, Wayne E. Clarke, Uday S. Evani, Marta S. Byrska‐Bishop, Anna O. Basile, Alexi Runnels, Rajeeva O. Musunuri, Giuseppe Narzisi, Hui Wang, Kelley M. Faber, Alison M. Goate, Brad F Boeve, Carlos Cruchaga, Margaret Pericak‐Vance, Jonathan L Haines, Roger N Rosenberg, Debby W Tsuang, Diones Rivera Mejia, Martin Medrano, Rafael A. Lantigua, Robert Sweet, David A. A. Bennett, Robert S. Wilson, Tatiana M. Foroud, Wan‐Ping Lee, Gerald D. Schellenberg, Clifton L. Dalgard, Richard Mayeux, Michael Zody, Badri N. Vardarajan

**Affiliations:** ^1^ The Gertrude H. Sergievsky Center, College of Physicians and Surgeons, Columbia University, New York, NY, USA; ^2^ The Taub Institute for Research on Alzheimer's Disease and the Aging Brain, Vagelos College of Physicians & Surgeons, Columbia University, New York, NY, USA; ^3^ Department of Neurology, Vagelos College of Physicians and Surgeons, Columbia University, and the New York Presbyterian Hospital, New York, NY, USA; ^4^ New York Genome Center, New York, NY, USA; ^5^ Department of Pathology and Laboratory Medicine, Perelman School of Medicine, University of Pennsylvania, Philadelphia, PA, USA; ^6^ Penn Neurodegeneration Genomics Center, Department of Pathology and Laboratory Medicine, Perelman School of Medicine, University of Pennsylvania, Philadelphia, PA, USA; ^7^ National Centralized Repository for Alzheimer's Disease and Related Dementias (NCRAD), Indianapolis, IN, USA; ^8^ Indiana University School of Medicine, Indianapolis, IN, USA; ^9^ Department of Medical and Molecular Genetics, Indiana University School of Medicine, Indianapolis, IN, USA; ^10^ Ronald M Loeb Center for Alzheimer's Disease, Department of Genetics & Genomic Sciences, Icahn School of Medicine at Mount Sinai, New York, NY, USA; ^11^ Department of Genetics and Genomic Sciences, New York, NY, USA; ^12^ Department of Neurology, Mayo Clinic, Rochester, MN, USA; ^13^ Washington University School of Medicine, St. Louis, MO, USA; ^14^ John P. Hussman Institute for Human Genomics, Miller School of Medicine, University of Miami, Miami, FL, USA; ^15^ Case Western Reserve University School of Medicine, Department of Population & Quantitative Health Sciences, Cleveland Institute for Computational Biology, Cleveland, OH, USA; ^16^ Department of Neurology, UTSouthwestern Medical Center Dallas, Dallas, TX, USA; ^17^ Geriatric Research, Education, and Clinical Center, VA Puget Sound Health Care System, Seattle, WA, USA; ^18^ CEDIMAT, Plaza de la Salud, Santo Domingo, Santo Domingo, Dominican Republic; ^19^ Universidad Pedro Henríquez Urena, Santo Domingo, Santo Domingo, Dominican Republic; ^20^ Pontificia Universidad Catolica Madre y Maestra, Santiago, Dominican Republic; ^21^ Department of Medicine, Vagelos College of Physicians & Surgeons, Columbia University, New York, NY, USA; ^22^ Department of Psychiatry and Neurology, University of Pittsburgh, Pittsburgh, PA, USA; ^23^ Rush Alzheimer's Disease Center, Rush University Medical Center, Chicago, IL, USA; ^24^ Indiana University School of Medicine/NCRAD, Indianapolis, IN, USA; ^25^ Penn Neurodegeneration Genomics Center, Perelman School of Medicine, University of Pennsylvania, Philadelphia, PA, USA; ^26^ Department of Anatomy, Physiology, and Genetics, Uniformed Services University of the Health Sciences, Bethesda, MD, USA

## Abstract

**Background:**

Alzheimer's disease (AD) exhibits significant genetic heterogeneity, with no single model fully explaining its inheritance pattern. Structural variants (SVs) may contribute to the missing heritability of AD, particularly in disease pathology and familial AD, which remain largely unexplored.

**Method:**

Structural variants (SVs) were analyzed in unrelated 1,162 individuals from the ROSMAP study (687 cognitively normal (CN), 475 AD patients), as well as 197 Non‐Hispanic White (NHW) families (926 individuals, 58.5% with AD) and 214 Caribbean Hispanic (CH) families (1,340 individuals, 59.17% with AD). Large insertions and deletions were identified using Manta, Absinthe, and MELT from whole‐genome sequencing, supplemented by Sniffles2 and external SV call sets. This approach generated a unified VCF file containing 45,251 insertions and 76,566 deletions. After filtering for missingness rate and allele counts, a total of 9,290 insertions and 9,529 deletions were retained for analysis. Genome‐wide SV analyses were performed with AD‐related metrics in ROSMAP, adjusting for age, sex, and principal components, and validated in an independent ADSP replication cohort comprising 29,055 unrelated individuals (17,110 CN and 11,945 AD patients).

**Result:**

We identified nine SVs (one insertion and eight deletions) segregating in AD families. An insertion near *PDCD10* in NHW families reached genome‐wide significance (*p* <5×10^‐8^) for AD risk in the replication cohort. Two deletions in *KIF26B* and *SPHKAP* were found in both CH and NHW families, while five appeared only in NHW and one in CH. All eight deletions showed genome‐wide significance for AD risk. SV GWAS on AD pathologies in ROSMAP revealed an insertion in *INPP4B* with the strongest association to global brain pathology (β=0.19; *p* = 2.32×10^‐5^), also linked to pathological AD, diffuse plaques, and Braak staging. A deletion near *ADGRB1* (β=1.53; *p* = 5.05×10^‐6^) was associated with neuritic plaques, and another near *ARMC2* (β=1.66; *p* = 2.17×10^‐6^) was linked to diffuse plaques. All three SVs associated with AD (*p* <5×10^‐6^) in replication.

**Conclusion:**

Our findings reveal numerous SVs segregating in familial AD across both CH and NHW families, as well as additional SVs linked to sporadic AD. Prioritizing these SVs based on their potential effects on gene function and expression will provide deeper insight into their contributions to both familial and sporadic AD.

